# Readiness for Interprofessional Learning Among Dental and Healthcare Students in a Geriatric Education Module: A Pre‐Professional Study

**DOI:** 10.1155/ijod/2027115

**Published:** 2026-07-13

**Authors:** M. Imran Pasha, Rashmi Jain, Umme Amarah

**Affiliations:** ^1^ Department of Public Health Dentistry, Yenepoya Dental College, Yenepoya (Deemed to be University), Mangaluru, Karnataka, India, yenepoya.edu.in; ^2^ Department of Ophthalmology, Yenepoya Medical College, Yenepoya (Deemed to be University), Mangaluru, Karnataka, India, yenepoya.edu.in; ^3^ Department of Oral Medicine and Radiology, Yenepoya Dental College, Yenepoya (Deemed to be University), Mangaluru, Karnataka, India, yenepoya.edu.in

**Keywords:** collaborative practice, dental education, dentistry, geriatrics, interprofessional education

## Abstract

**Introduction:**

Interprofessional education (IPE) fosters collaboration among healthcare students, preparing them for coordinated, patient‐centered care. Geriatric care, requiring multidisciplinary input, offers an ideal framework for interprofessional learning. This study assessed the readiness for interprofessional learning among students of Yenepoya (Deemed to be University) who participated in the geriatric IPE program (GIEP) workshop.

**Methods:**

A pre and postintervention study was conducted among undergraduate students from medicine, nursing, physiotherapy, and allied health sciences. Participants completed the 19‐item Readiness for Interprofessional Learning Scale (RIPLS), which measures four domains: teamwork and collaboration, positive professional identity, negative professional identity, and roles and responsibilities. Responses were rated on a five‐point Likert scale. Data were analyzed using descriptive statistics Mann–Whitney *U* test to compare pre‐ and post‐workshop scores.

**Results:**

Seventy students completed the pre‐workshop and 48 completed the post‐workshop assessments. The mean total RIPLS score decreased slightly from 72.91 ± 7.84 to 71.19 ± 10.08. Subscale comparisons revealed minor, nonsignificant changes across all domains (*p* > 0.05), except in roles and responsibility where the difference was significant.

**Conclusion:**

Students exhibited a high baseline readiness for interprofessional learning, reflecting positive attitudes toward teamwork and collaboration. Although no statistically significant improvement was observed following the GIEP workshop, the intervention reinforced interprofessional values. Long‐term and integrated IPE initiatives are recommended to strengthen collaborative competencies and measure sustained changes in readiness.

## 1. Introduction

The global demographic profile is undergoing an unparalleled transformation, marked by a rapidly aging population. The population of individuals aged 60 years and older is anticipated to double, approaching 2.1 billion by 2050 [1]. This change has caused many problems for modern healthcare systems. Older patients often have multiple health problems and cognitive impairments and take many medications, which means that treatment needs to be coordinated between different professionals instead of being done separately [2]. The World Health Organization (WHO) has called for a shift from isolated professional practice to a collaborative, patient‐centered approach to meet these complex needs [3]. In order to enhance collaboration and care quality, students from two or more professions learn “with, from, and about each other” through interprofessional education (IPE) [4]. A “collaborative‐ready” healthcare workforce that can handle the interdependencies of complicated clinical cases is developed through IPE [5]. The management of an elderly patient with systemic health issues and oral care needs, such as drug‐induced xerostomia or diabetes linked to periodontitis, cannot be handled by a single practitioner. This illustrates the significance of IPE in geriatric care. To maximize health outcomes, these call for the combined expertise of physiotherapists, pharmacists, and dentists [6]. Several strong educational and social theories, such as Malcolm Knowles’ Adult Learning Theory (Andragogy), which contends that adult learners are motivated by the immediate relevance and practical application of their education, support the efficacy of IPE [7]. IPE makes this possible by putting students in real‐world, case‐based situations, where cooperation is essential. Additionally, knowledge is a social product created through interaction and shared experiences, according to Social Constructivism [8]. Students in the IPE setting create a shared professional reality that recognizes the importance of other disciplines rather than merely learning clinical facts. This is in line with the contact theory, which contends that interactions between various professional groups can lessen prejudice and stereotypes as long as the learners have institutional support and similar objectives [9]. Furthermore, the “learning‐by‐doing” cycle, which is crucial for students to move from theoretical knowledge to clinical interprofessional competence, is emphasized by the experiential learning theory [10]. IPE’s integration into undergraduate curricula, especially in dental education, is still lacking in many areas despite its obvious theoretical advantages [11]. Dental students have historically been kept apart from other health streams, creating a “professional silo” effect that may make it more difficult to provide care for elderly patients who are physically weak or have compromised health [12]. Although introductory IPE modules have been implemented in many institutions, there is insufficient empirical data to determine how these interventions alter students’ “readiness” for collaborative practice over time [13]. The Readiness for Interprofessional Learning Scale (RIPLS) can be used to measure the developmental state of readiness for interprofessional learning [14]. Previous research, however, has produced contradictory findings; some document a phenomenon of “recalibration,” in which students’ scores actually decline after the intervention, while others report an increase in readiness [15]. The stages of professional identity formation, where students transition from an idealized conception of “teamwork” to a more realistic, albeit conservative, understanding of professional boundaries and hierarchies encountered in actual clinical settings, are frequently blamed for this decline [12, 15]. Understanding how IPE affects dental, pharmacy, and physiotherapy students in the particular setting of geriatric care is a major research gap. The majority of the literature currently in publication concentrates on nursing and medical students, frequently ignoring the vital role that pharmacological management and oral health play in the elderly [11]. This study aims to address this gap by evaluating the changes in readiness among healthcare professional students participating in the geriatric IPE program (GIEP) module.

## 2. Methodology

GIEP module workshop was conducted amongst the students of the Yenepoya (Deemed to be University); the flyers were distributed to the constituent colleges prior to the program, and participants were asked to register voluntarily for the workshop conducted on 23rd January 2025. This was a pre–postintervention study conducted to evaluate the impact of a workshop‐based GIEP on the attitudes and readiness of healthcare professional students toward collaborative practice. The sample size for this study was computed using G power to detect the effectiveness of the “Geriatrics IPE” Program Module Amongst the students at Yenepoya (Deemed to Be University). The sample size for this study was calculated at 1% level of significance and 99% power, to detect the specified effect size is 41, keeping in mind the loss to follow‐up and other factors, a total of 70 students were invited to participate voluntarily. The RIPLS was utilized as the primary tool for the study. The scale consists of 19 items scored on a 5‐point Likert scale (1 = strongly disagree to 5 = strongly agree). The instrument was administered twice: once immediately before the workshop (pre‐test) and again following the completion of the module (post‐test). Descriptive statistics were utilized to summarize the demographic profile of the participants. Continuous variables (age and RIPLS scores) were expressed as mean ± standard deviation (*M* ± SD), while categorical variables (gender, stream of study, and IPE awareness) were presented as frequencies and percentages. The internal consistency and reliability of the 19‐item RIPLS instrument were assessed using Cronbach’s alpha (*α*). Prior to inferential testing, the distribution of RIPLS scores was evaluated for normality using the Shapiro–Wilk test. The Mann–Whitney *U* test was selected as the primary inferential tool to compare the median differences between the two independent cohorts. The relationship between chronological age and RIPLS scores was evaluated using Pearson’s correlation coefficient (*r*) to determine if maturity acted as a confounding variable. The effect size was measured using Cohen’s *d*, which quantifies the magnitude of the difference between the two groups. In this study, unequal response analysis (also known as an independent or unmatched samples analysis) was done includes all participants who completed the questionnaires at either time point, rather than restricting the study to only those who completed both to reduce the responder bias and to also reflect the real world impact of the program.

## 3. Results

**Figure 1:**
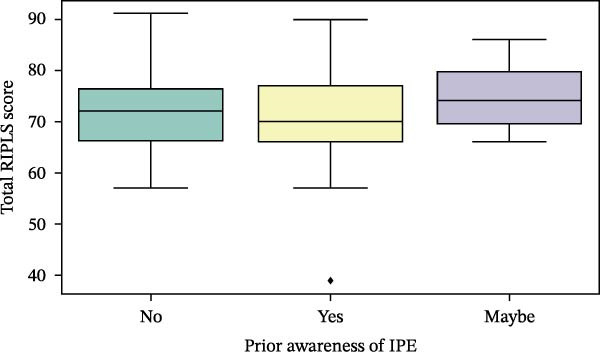
Total RIPLS scores compared to the awareness of IPE among the study cohorts.

**Figure 2:**
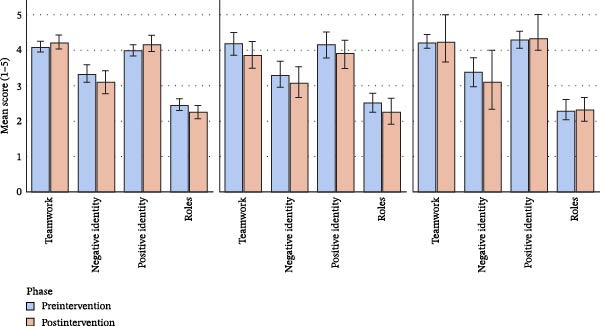
RIPLS subscale scores with awareness of IPE among the study cohorts.

The unequal response analysis (also known as an independent or unmatched sample analysis) has been done for data which includes all participants who completed the questionnaires at either time point, rather than restricting the study to only those who completed both. This analysis provided a broader perspective of the student response to GIEP Modules. Table 1 shows that both pre–post participants were of the mean age 23 years,majority were female participants, and dental, physiotherapy, and pharmacy participants were part of the workshop. Table 2 results shows that the readiness for teamwork remained high and stable across the cohort, with no significant difference between the total pre and post groups (*p* = 0.736). Statistically significant decrease in the “Roles and Responsibilities” score was found in the study cohort (*p* = 0.018). The professional Identity subscales like negative professional identity has seen a score decrease (from 10.04 to 9.31) and positive professional identity subscale remained almost identical (16.30 vs. 16.33) and were not statistically significant, respectively. Table 3 shows that preintervention, both genders showed high levels of readiness, with males scoring slightly higher on average score (73.76) compared to females (72.49). Postintervention males showed an increase in their mean readiness score (73.76–75.94) when compare to females, who showed a decrease in mean scores (72.49–68.58). Table 4 shows that physiotherapy students showed a substantial increase in their readiness scores (70.55–79.71). Both dental (71.89–68.70) and pharmacy (74.31–71.71) students saw a decrease in mean scores. Table 5 shows that there was a weak positive correlation (*r* = 0.211) between age and RIPLS scores preintervention and the correlation essentially decreased (*r* = −0.02, *p* = 0.895) postintervention. Figure 1 shows that there is no statistically significant difference in readiness scores based on prior awareness. Figure 2 shows that prior awareness of IPE saw the most significant decrease in scores postintervention (73.50–68.55) and those who had never heard of IPE before the program showed remarkable stability and even a slight improvement in teamwork (36.82–38.00). Roles and responsibility decreased across all spectrum, and also the positive identity subscale remained stable. The GIEP intervention demonstrated a moderate effect on students’ perceptions of roles and responsibilities (Cohen’s *d* = −0.43) and a small overall effect on total interprofessional readiness (Cohen’s *d* = −0.20). These results suggest that the workshop was most effective in clarifying the complexities of professional boundaries in geriatric care. The internal consistency of the RIPLS was verified using Cronbach’s alpha (*α* = 0.921).

**Table 1:** Demographic details of the study participants pre and postintervention.

Characteristic	Preintervention (*N* = 70)	Postintervention (*N* = 48)
Age (mean ± SD)	23.3 ± 1.4	23.4 ± 2.0
Gender^a^: female	42 (60.0%)	34 (70.8%)
Gender^a^: male	27 (38.6%)	13 (27.1%)
Dental students	30 (42.9%)	28 (58.3%)
Pharmacy students	23 (32.9%)	12 (25.0%)
Physiotherapy students	17 (24.3%)	8 (16.7%)

^a^One student did not respond to gender category pre and postintervention.

**Table 2:** Pre–post readiness for interprofessional learning among health care professions students at Yenepoya (Deemed to be University).

RIPLS subscale	Pre mean (SD)	Post mean (SD)	*p*‐Value
1. Teamwork and collaboration	37.23 ± 4.39	36.69 ± 6.49	0.736
2. Negative professional identity	10.04 ± 2.43	9.31 ± 2.76	0.129
3. Positive professional identity	16.30 ± 2.25	16.33 ± 3.07	0.577
4. Roles and responsibilities	9.34 ± 1.10	8.85 ± 1.17	0.018
Total RIPLS score	72.91 ± 7.84	71.19 ± 10.08	0.233

**Table 3:** Distribution of pre and post RIPLS score by gender.

Study phase	Gender	Count (*N*)	Mean score	Std. deviation (SD)
Preintervention	Female	39	72.49	6.89
	Male	29	73.76	9.18
Postintervention	Female	31	68.58	9.4
	Male	17	75.94	9.78

**Table 4:** Distribution of pre and post RIPLS score by stream of study.

Study phase	Stream of study	Count (*N*)	Mean score	Std. deviation (SD)
Preintervention	Dental student	28	71.89	7.75
	Pharmacy student	29	74.31	6.45
	Physiotherapy student	11	70.55	10.97
	Nursing student^a^	1	81	N/A
Postintervention	Dental student	27	68.7	9.74
	Pharmacy student	14	71.71	7.01
	Physiotherapy student	7	79.71	12.85

^a^Single respondent; data included for completeness but not statistically analyzed.

**Table 5:** Correlation of RIPLS score by age.

Phase	*N*	Pearson *r*	*p*‐Value (Pearson)	Spearman *ρ*	*p*‐Value (Spearman)
Preintervention	67	0.211	0.087	0.082	0.508
Postintervention	47	−0.02	0.895	−0.044	0.767

## 4. Discussion

GIEP as a workshop‐based approach aimed for collaborative learning for geriatric patients amongst dental, pharmacy, and physiotherapy students. The RIPLS, developed by Parsell and Bligh [14], was utilized in the study. The high level of readiness before the intervention in all streams is in line with the idea that working together across professions is necessary to improve healthcare outcomes [1, 4]. This is important in geriatrics because multimorbidity is common and treatment plans need to be thorough [2]. Our results indicated a statistically significant reduction in the roles and responsibilities subscale (*p* = 0.018). Even though a drop might seem strange, it probably means that people are moving from idealistic views to a more realistic understanding of how complicated geriatric teamwork can be. This phenomenon, known as a reality check, occurs when students become more aware of the difficulties of role blurring and coordination after actually working with a team [15]. The GIEP Program modules were grounded in the experiential learning theory [10] and adult learning principles [7], which emphasize that change occurs through active experience and reflection. Utilizing the curricular framework within a theoretical framework for engaged learning [8] compelled students to transcend theoretical awareness and achieve practical engagement, thereby directly influencing professional identity formation [12]. Our findings indicated a notable transformation in the professional identity subscale, implying that the GIEP successfully addressed the compartmentalized identities frequently observed among dental and other health professional students [6, 11]. As the students worked together, they started to deal with the tension between their own professional skills and the needs of the geriatric patient [9, 12]. The difference in scores between streams where physiotherapy students got better and dental and pharmacy students got worse can be explained by the different levels of traditional autonomy in these fields. Historically, dental education has emphasized clinical independence [6], while pharmacy has concentrated on particular pharmacotherapeutic roles. In contrast, physiotherapy is often naturally integrated into multidisciplinary geriatric rehabilitation [15]. The GIEP served as a narrative scoping experience for these students [5], highlighting that in geriatric care, oral health and medication management cannot be isolated from functional mobility [11]. The lack of correlation between age and RIPLS scores indicates that the readiness for collaboration is a matter of educational exposure rather than age maturity. Furthermore, the fact that prior awareness of IPE did not prevent the postintervention decrease in scores reinforces findings by Leung et al. [13] and Teuwen et al. [3] specifically, that student‐initiated or classroom awareness is not a substitute for formal, structured interprofessional programs. For IPE to enhance interest in treating older people effectively, it must be embedded as a longitudinal experience that allows for continuous reflection [3, 13]. The positive professional identity subscale remained identical pre and postintervention (16.30 ± 2.25 vs.16.33 ± 3.07), which can be due to the fact that professional identity is an internalized construct that develops through repeated socialization and longitudinal exposure to professional norms [12]. The ceiling effect, which is observed in this cohort, is also the reason for the above effect. A “Spiral curriculum” approach where geriatric IPE concepts are revisited and deepened at key milestones of dental training can be integrated into the existing curriculum [13]. Limitation of the study is that current study utilized a single‐center, unmatched design, which serves as a foundational baseline for future longitudinal, multicenter studies utilizing paired data tracking.

## 5. Conclusions

The RIPLS score in the GIEP module workshop shows its dynamic nature and the module had a positive impact on roles and responsibilities and maintained positive professional identity postintervention.

## Funding

This study was funded by the Yenepoya (Deemed to be University).

## Conflicts of Interest

The authors declare no conflicts of interest.

## Data Availability

The data that support the findings of this study are available from the corresponding author upon reasonable request.
